# Epidemiological and Phylogeographic Study of Equid Herpesviruses in Tunisia

**DOI:** 10.3390/pathogens11091016

**Published:** 2022-09-05

**Authors:** Chaima Badr, Oussama Souiai, Marwa Arbi, Imen El Behi, Mohamed S. Essaied, Ines Khosrof, Alia Benkahla, Ahmed Chabchoub, Abdeljelil Ghram

**Affiliations:** 1 Laboratory of Epidemiology and Microbiology Veterinary (LR19IPT03), Institute Pasteur of Tunis, University Tunis El Manar, Tunis 1002, Tunisia; 2 Laboratory of Bioinformatics, Biomathematics and Biostatistics (LR16IPT09), Institute Pasteur of Tunis, University Tunis El Manar, Tunis 1002, Tunisia; 3 National School of Veterinary Medicine, Sidi Thabet, University of Manouba, La Manouba 2010, Tunisia; 4 Laboratory of Viruses, Vectors and Hosts (LR20IPT10), Institute Pasteur of Tunis, University Tunis El Manar, Tunis 1002, Tunisia

**Keywords:** equine, herpesvirus, EHV1, EHV2, EHV5, gB, epidemiology, phytogeography, Tunisia

## Abstract

Equid herpesvirus (EHV) is a contagious viral disease affecting horses, causing illness characterized by respiratory symptoms, abortion and neurological disorders. It is common worldwide and causes severe economic losses to the equine industry. The present study was aimed at investigating the incidence of EHVs, the genetic characterization of Tunisian isolates and a spatiotemporal study, using 298 collected samples from diseased and clinically healthy horses. The global incidence of EHV infection was found to be about 71.81%. EHV2 and EHV5 were detected in 146 (48.99%) and 159 (53.35%) sampled horses, respectively. EHV1 was detected in 11 samples (3.69%); EHV4 was not detected. Co-infections with EHV1-EHV2, EHV1-EHV5 and EHV2-EHV5 were observed in 0.33%, 1.34% and 31.54% of tested horses, respectively. Phylogenetic analyses showed that gB of EHV2 and EHV5 displays high genetic diversity with a nucleotide sequence identity ranging from 88 to 100% for EHV2 and 97.5 to 100% for EHV5. Phylogeography suggested Iceland and USA as the most likely countries of origin of the Tunisian EHV2 and EHV5 isolates. These viruses detected in Tunisia seemed to be introduced in the 2000s. This first epidemiological and phylogeographic study is important for better knowledge of the evolution of equid herpesvirus infections in Tunisia.

## 1. Introduction

Equid herpesviruses (EHV) are ubiquitous enveloped DNA viruses of the family *Herpesviridae*. They have a major economic and health impact on all sectors of the horse industry worldwide. They have direct clinical effects on the horse, including respiratory disease, abortion and paralysis, as well as on the horse industry, including horse movement for competition and breeding [1].

To date, all nine described EHV species/subtypes belong to either the *Alphaherpesvirinae*, including (EHV1, EHV3, EHV4, EHV6, EHV8 and EHV9) or *Gammaherpesvirinae*, including (EHV2, EHV5 and EHV7) subfamilies [2]. The most studied EHVs are the alphaherpesviruses EHV1 and EHV4 since they pose the most serious health risks [3]. EHV4 was recently incriminated in outbreaks of respiratory disease in Germany, Romania and China [4,5,6]. EHV1 is mostly implicated in abortion, neonatal and perinatal death and neurological disease [3]. Recently, an EHV1 outbreak, originating at an International Horse Jumping event held in Valencia (Spain), leading to the cancellation of sport horse events, was rapidly spread to over 30 premises in different geographical areas of Belgium, Denmark, Spain, France, Germany, Italy, Qatar, Slovakia, Sweden and Switzerland and causing 18 dead horses [7].

Equid gammaherpesviruses are widespread and quite prevalent in equine populations. They have been associated with multiple disease processes, but it is still unclear whether they are an inciting cause of the disease, a co-factor or innocent bystanders [8]. EHV5 is associated with the development of equine multinodular pulmonary fibrosis (EMPF), lymphoproliferative disorders, dermatitis, systemic granulomatous disease and ocular disease [9,10,11,12,13], while EHV2 is associated with upper respiratory tract disease, pharyngitis, kerato-conjunctivitis, poor performance and idiopathic systemic granulomatous disease (ISGD), known as equine sarcoidosis [14,15,16,17,18,19,20,21]. Furthermore, both viruses have been isolated from aborted fetuses and placentas and seem to be associated with abortions [22,23].

All equid herpesviruses establish lifelong latency in infected horses. The alphaherpesviruses establish latency in the sensory neurons or lymphocytes of their hosts [24] while gammaherpesviruses establish latency in lymphoid tissue and peripheral blood leucocytes [8]. Viral reactivation and shedding can then occur at any time following various stress exposures, such as weaning, foaling, castration, transport and overworking 

In Tunisia, the number of horses is about 26,000 heads, of which 14,000, 6000, 5000, and 1000 are Arab-Barb, Barb, Arabian and English Thoroughbred breeds, respectively. There are also 40,000 male and female mules [25]. All these breeds are mainly used for breeding, leisure, race events and export. EHV serological evidence was first reported in 1985 in Tunisia [26], and since then, few epidemiological investigations have indicated that EHVs are prevalent amongst the Tunisian equine population [27,28], even though the epidemic status of EHV is of concern.

The aim of this study was to investigate the incidence of EHVs among the horse population, the genetic characterization of EHV2 and EHV5 Tunisian isolates and the spatiotemporal study of equid gammaherpesviruses using Bayesian phylodynamics analyses, to report their origin and spread

## 2. Results

### 2.1. Virus Detection by PCR

The results of PCR tests, realized on 298 swabs and organs samples, showed a global EHV detection prevalence of 71.81% (214/298) for at least one virus amongst the four considered equid herpesviruses. The most prevalent was infection with EHV5 with 53.35% (159/298), followed by EHV2 with 48.99% (146/298) and EHV1 with 3.69% (11/298). None of the horses were positive for EHV4. Furthermore, approximately 31.54% (94/298) of detected horses were positive for both EHV2 and EHV5; the remaining horses were positive for only EHV2 with 16.44% (49/298) or EHV5 with 20.13% (60/298) (Table 1).

The prevalence of horses positive for EHV5 varied among age groups (Table 2). Thus, horses under five years old were more likely to be positive for EHV5 than other age groups (*p*-value = 0.0057). On the contrary, horses over 10 years old seemed to be less affected by EHV2 (*p*-value = 0.0329) than other age groups. However, there was no significant correlation between EHV1 infection and age groups. Considering gender, males were more likely to be infected by EHV5 (*p*-value = 0.0017) than females (*p*-value = 0.0363).

The prevalence of positive horses varied among breeds. For this, EHV1 infection was more prevalent in BAB horses (*p*-value = 0.0092) than EHV2 (*p*-value = 0.0108) and EHV5 (*p*-value = 0.0150) than other breeds. On the other hand, Arabian thoroughbred horses were most likely to be positive for EHV2 (*p*-value = 0.024) than EHV1 or EHV5.

In relation to horse activities, racehorses seemed to be more affected by EHV1 (*p*-value = 0.0246) or EHV2 (*p*-value = 0.0009) than other activity groups. However, Breeders were more likely to be positive for EHV1 (*p*-value = 0.0015) and EHV5 (*p*-value = 0.0463) than other groups.

Concerning the seasons, EHV1 (*p*-value = 0.0001), EHV2 (*p*-value = 0.0226) and EHV5 (*p*-value = 0.0441) were less prevalent in horses sampled in autumn than those sampled in any other times of the year.

### 2.2. Association of EHVs Infection and Presence of Clinical Signs

Table 2 show the association between EHV1, EHV2 or EHV5 infections and the expression of clinical signs such as respiratory difficulties, nasal discharge, dyspnea and coughing. By comparing diseased and healthy groups, a significant proportion of EHV1 infected equids, 54.5%, showed clinical respiratory signs (*p*-value = 0.0214); in addition, 36.4% of EHV1-infected horses were clinically healthy (4/43), and the clinical status of the remaining 9.1% of horses does not have any available data. However, there was no significant relationship between EHV2 or EHV5 infected animals showing clinical signs and those not showing any clinical signs but positive for EHV2 or EHV5.

### 2.3. Molecular Characterization and Phylogenetic Analysis

#### 2.3.1. EHV1 and EHV4

It is worth noting that no EHV4 was isolated during this study, and no phylogenetic analysis was realized. Furthermore, characterization of EHV1 isolates in various field samples (vaginal and nasal swabs, aborted organs) using PCR has allowed the identification of 11 positive samples, showing relatively high Ct values and an incidence of 3.69% (Table 1 and Table 2). Unfortunately, passages in cell cultures (Vero cell line) and SPF embryonated eggs did not allow sufficient virus growth for gene sequencing and subsequent analyses. The sample quality was not as good as expected since they were sometimes received in bad conditions or relatively late after abortion or clinical manifestations.

#### 2.3.2. EHV2

The partial nucleotide and amino acid sequences of the gB gene of 18 EHV2 isolates were compared with each other and with those from GenBank. The Tunisian EHV2 isolates showed genetic diversity with nucleotide sequence identities between 88 to 100% and amino acid sequence identities that range from 90.2 to 100% amongst each other. As compared to sequences from GenBank, gB genes of Tunisian EHV2 isolates shared 92.13–100% identity with isolates from Australia, Switzerland, Iceland and the UK.

#### 2.3.3. EHV5

The partial nucleotide and amino acid sequences of the gB gene of 23 EHV5 isolates were compared with each other and with those from GenBank. The degree of identity between the Tunisian EHV5 isolates ranged from 97.5 to 100% at the nucleotide level and 96.9 to 100% at the amino acid level, as compared to each other. Furthermore, the gB genes from EHV5 shared a high degree of identity (91.30–100%) with isolates from China, Korea, Australia, Iceland, the USA, Italy and Ethiopia.

### 2.4. Spatiotemporal Dynamics of Herpesvirus Isolates

**Figure 1:**
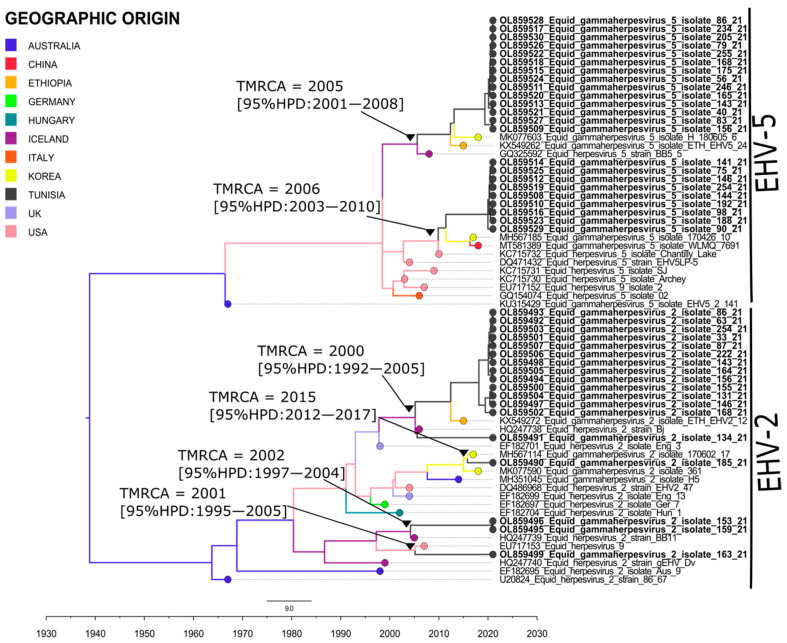
Time scaled Maximum Clade Credibility (MCC) tree representing geographic origins of equid herpesviruses 2 and 5 isolated in Tunisia. Tree colors reflect the most common ancestral geographic origin of tree nodes and branches. TMRCA and their 95% HPD of Tunisian isolates are indicated in the tree. Tunisian isolates identified in this study are indicated in Bold.

For EHV5, molecular clock analysis indicated that the Time to the Most Recent Common Ancestor (TMRCA) of the Tunisian clades is around 2005–2006. However, the TMRCA of EHV2 was around 2000–2002 and 2015. These findings showed that the 2000s period has probably witnessed the first introduction of equid gammaherpesviruses EHV2 and EHV5 in Tunisia. According to the branch colors of the MCC tree (Figure 1), only Iceland presents the most recent common ancestor for EHV5 Tunisian clades, whereas both the USA and Iceland are the most recent common ancestral geographic origin for EHV2 strains of Tunisia.

**Figure 2:**
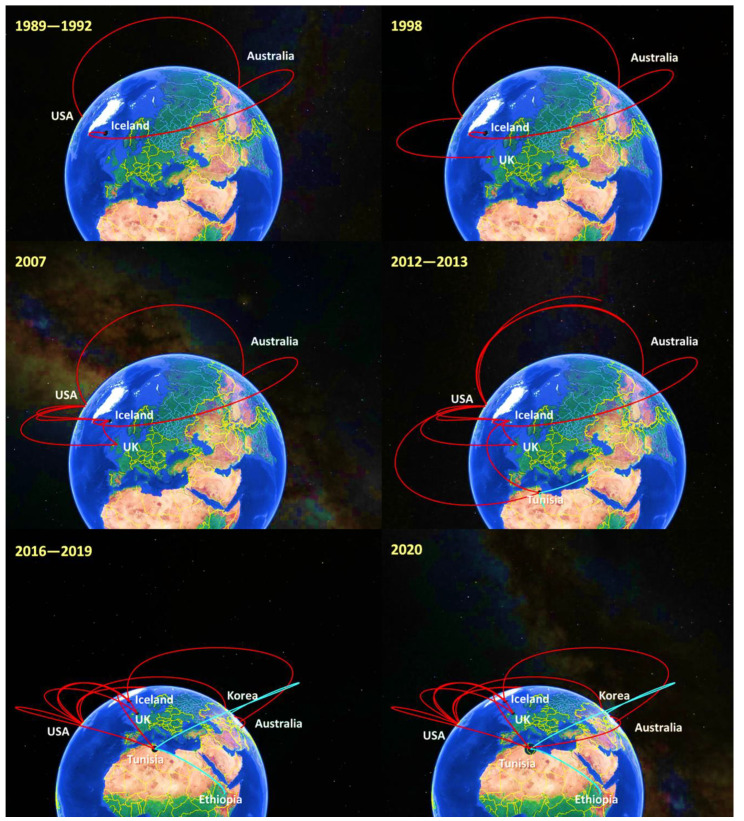
Spatiotemporal dynamics tracking the historical origin of equid herpesviruses 2 and 5 isolated in Tunisia. Lines connecting between countries represent branches in the MCC tree. Lines in blue indicate the virus transmission from Tunisia to other countries.

To have a better knowledge on the ancestral history of equid gammaherpesviruses isolated from Tunisia, the MCC tree was used to construct a world geographic transmission network. Spatiotemporal dynamic analysis showed that our isolates of EHV2 and EHV5 genetically originated from gammaherpesviruses strains, circulating during 1989 among Australian horses. Since then, horses from Iceland and the USA have been infected with gammaherpesviruses originating from Australia. By the end of the 1990s, gammaherpesviruses were transmitted from the USA to the UK. In 2007, we observed that Iceland became a virus accumulation point where horses are affected by several gammaherpesviruses from the USA and the UK. During 2012–2013, strains circulating in the USA and Iceland were at the origin of gammaherpesviruses cases in Tunisia. From 2016 to 2019, we noticed that Korean and Ethiopian horses were infected by gammaherpesviruses originating from Tunisia. Finally, gammaherpesviruses continued to occur in Tunisia through horses’ contamination by Korean strains during 2020 (Figure 2) (Supplementary Files, SF1).

**Figure 3:**
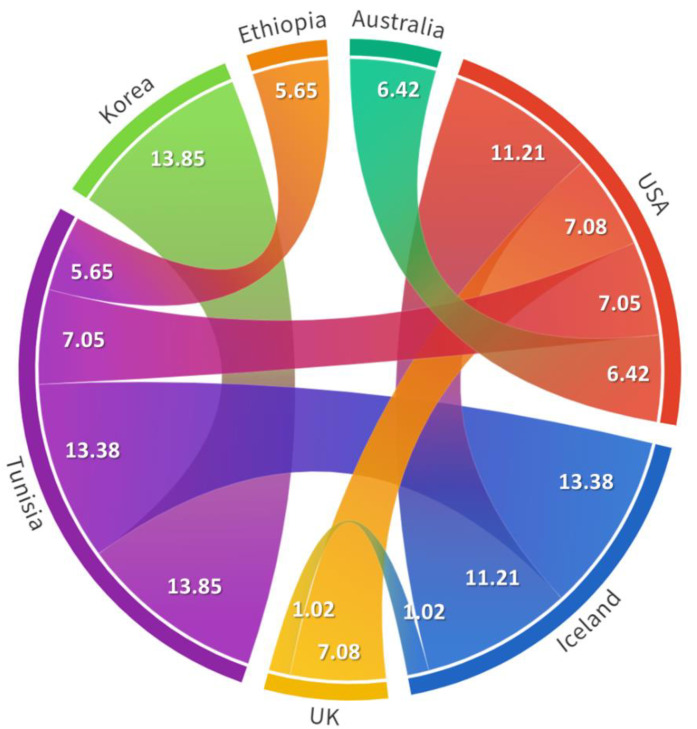
Chord diagram representing the identified herpesvirus transitions between countries with their Bayes Factor (BF). Chord width is proportional to BF. Bayes Factors for each transition are indicated in the figure. A transition with BF > 3 is statistically supported.

To provide statistical support for our spatiotemporal dynamics’ findings, we calculated the Bayes factor (BF) for each identified transition and found that all of them are statistically supported (BF > 3) except the UK–Iceland transition, showing a BF equal to 1.02. The transitions having the highest Bayesian support (BF > 10) were those linking Tunisia to Korea (BF = 13.85), Iceland to Tunisia (BF = 13.38) and the USA to Iceland (BF = 11.21) (Figure 3).

## 3. Discussion

Equid herpesvirus outbreaks are nothing new, but their effects can still be dramatic, shutting down equine events, obstructing horse transport across state lines and causing panic in corners of the industry, as EHVs and especially alphaherpesviruses threaten equine health worldwide. Various equid herpesviruses, EHV1, 2, 4 and 5, are regularly detected in infected animals, and valuable athletic horses can develop upper respiratory difficulties leading to exercise intolerance, abnormal respiratory sounds, poor performance, abortion or neurological disorders.

Our results showed incidences of 48.99% and 53.35% for EHV2 and EHV5 gammaherpesviruses, respectively. However, such incidences were much higher than those seen for EHV1 and EHV4 alphaherpesviruses, with 3.69% and 0%, respectively. These results are in accordance with another study, reporting a low frequency of alphaherpesviruses (<10%) and a high incidence of gammaherpesviruses (0–100%) [29].

The high rates of EHV2 and EHV5 infections among Tunisian horses were consistent with data reported from Australia, Argentina, Brazil, Canada, Ethiopia, Germany, Hungary, Iceland, Italy, Japan, New Zealand, Poland, Algeria, Serbia, Switzerland, Turkey, the United Kingdom, the United States and South Korea [23,30,31,32,33,34]. Indeed, EHV5 was detected with a higher frequency than EHV2, which is in agreement with other reports from Algeria [31], Australia [29], Turkey [35], Korea [36], China [6] and Ethiopia [32], but in contrast with other studies from Poland [37], Sweden, Hungary, the UK [38], New Zealand [39] and Iceland [40], where EHV2 was more commonly identified. These data indicated that the incidence of gammaherpesvirus infections varies amongst equine populations, depending on factors such as sampled animals and geographical locations. 

It is well known that horses that test positive for any EHV strain do not necessarily show any signs of illness, including nasal discharge, rapid breathing or fever. Indeed, EHV1 has not been easily detected in our tested samples, and only a few isolates were identified either in symptomatic or asymptomatic horses. These results are consistent with a recent study that reported high levels of EHV1 or EHV4 shedding in five clinically healthy horses [29]. In fact, reactivation of latent alphaherpesviruses infection is often associated with subclinical viral shedding [41]. In contrast, EHV2 and EHV5 are usually isolated from horses with or without respiratory symptoms. Furthermore, in a previous Tunisian study, only EHV2 and EHV4 were detected in horses with chronic respiratory disease, with incidences of 33.3% and 6.7%, respectively; neither EHV1 nor EHV5 was identified [28].

Interestingly, our study showed that EHV2 had been detected in apparently healthy horses, and the presence of EHV2 and EHV5 in horses clinically healthy might increase the risk of other possible infections by compromising host immunity, as previously reported [6,42]. However, a high proportion of horses with clinical signs and shedding of EHV5 was reported in a recent study [29]. Such an increased proportion of EHV5 shedding among diseased horses, attaining 86.2%, might reflect the contribution of EHV5 to the declared respiratory disease. In fact, EHV5 is usually associated with equine multinodular pulmonary fibrosis (EMPF), while it is often detected in both apparently healthy and diseased horses [43]. Alternatively, such virus shedding might have been reactivated as a consequence of the respiratory disease-associated inflammatory response.

In accordance to a recent Ethiopian study [32], the highest prevalence of EHV5 (23.1%) was detected in horses with respiratory signs, followed by EHV2 (20.0%), EHV4 (8.1%) and EHV1 (7.5%); however, EHV1 and EHV4 were never detected in healthy horses, while EHV2 and EHV5 were found with an incidence of 7.2% and 16.2%, respectively. More recently, nasal swab samples from Poland have shown higher incidences of EHV2 (77.2%) and EHV5 (47%) as compared to EHV4 (0.4%) [37]. Furthermore, EHV2 (2.3%) and EHV5 (2.6%) were only detected in genital swabs from healthy horses [22]. On the contrary, EHV1 (5.6%) and EHV4 (7.9%) were only detected in nasal swabs from horses suffering from respiratory diseases [35].

In the present study, dual or triple infections with EHV1 and EHV2, EHV1 and EHV5, EHV2 and EHV5 or EHV1, EHV2 and EHV5 were also detected, as reported in other studies [31,32,35,36,37,44,45]. In fact, shedding of multiple EHVs was detected in 14% of tested horses; four out of six of them (67%) being infected with alphaherpesviruses EHV1 or EHV4, along with another alpha- and/or gammaherpesviruses [43].

In relation to age groups, there were no significant differences seen for EHV1 infections. On the other hand, significant variations were observed for EHV2 and EHV5 infections amongst age groups. In fact, the highest prevalence of EHV2 and EHV5 were recorded at a rate of 43.8% in young (<5 years old) than in older horses (21.4%) [28]. This is in line with what has been reported in other studies, showing that young equids are at greater risk of developing clinical respiratory diseases associated with EHV infections [32,42,46,47,48]. Young foals are probably infected through direct contact with their dams during the first months of life, after which the virus is transmitted horizontally to contact foals [42,49]. Males were more likely to be positive for EHV5 than females, as was the case for the frequency of EHV2, as reported in other studies [28,36].

Our study showed that the proportion of EHV1, EHV2 and EHV5 positive horses varied with seasons. Thus, horses sampled in winter were more likely to be shedding EHV2 and EHV5 than those sampled at any other time, in contrast to the result of Stasiak et al. stating that horses sampled during springtime are more likely to shed EHV2 [37]. Such seasonality of EHV incidence appeared to be related, in Tunisia, to racehorses, in close relation to climatic changes, as cold winter has been identified as a stressor for EHV reactivation [50]. Furthermore, the majority of young horses are in contact, during their first years of life, with EHV1 and/or EHV2, responsible for epizootics mainly observed in autumn and winter, contrasting with subclinical or sometimes unapparent forms appearing in adults [51,52].

Of the various breeds included in this study, BAB horses seemed to be less infected with either EHV2 or EHV5 and more likely to be shedding EHV1 than other horse breeds; Barb horses represented the oldest population sampled (5–10 years). On the other hand, Arabian thoroughbred horses were more likely to be positive for EHV2, as they represent the youngest horse population studied (<5 years), supported by data reported by Stasiak et al. [37]. 

As horses are often latency infected, even at low levels, they are potentially considered at high risk of EHV infections and stress during transportation, training periods, race competitions or breeding periods following virus reactivation. For this, our results showed that racehorses, which are constantly under higher stress levels, are more likely to be positive for EHV1 than EHV2 and/or EHV5 than other activity groups. In Tunisia, horses are usually introduced into training centers at the age of 2 years in the spring of each year, and horse races are carried out almost year-round. 

Traveling effects on the incidence of equid herpesviruses are very important, knowing that athletic horses are the terrestrial mammals that travel the most after humans worldwide. They also represent an important element to be considered in the transmission of pathogens amongst equids and other species [53]. In this study, we found that 7 out of 10 horses examined after travel are positive for at least one herpesvirus, three co-infected with EHV2 and EHV5 and none infected with EHV1 and/or EHV4. Thus, our results indicate that transportation might lead to increased shedding, transmission and reactivation of EHV2 and EHV5, but not EHV1 or EHV4. Unlike previously reported data, focusing on the role of alphaherpesviruses transport-related disease, our research results suggest that investigations on gammaherpesviruses should not be dismissed, particularly given the fact that such viruses may encode suppressive immuno-modulators affecting host health [54]. Equid herpesviruses, similar to other herpesviruses, enter a latent state and may be reactivated, resulting in recurring disease, which is accompanied by virus shedding and transmission to other horses [55,56,57]. The risk of respiratory diseases affecting transported horses may increase following stress-associated immunosuppression, primarily by opportunistic bacterial proliferation and virus reactivation. In fact, 12 hours of transportation induces acute stress in horses, although viral replication is not observed [58]. Virus neutralizing (VN) antibody titers against EHV1 decreased temporarily in nasal secretions after transportation, suggesting that suppression of VN capacity of the nasal mucosa may contribute to the susceptibility to EHV1 after transportation stress [58].Finally, the study of Muscat et al. [54] reported the possible role of equid herpesvirus in the development of transport pneumonia. Surprisingly, in the latter study, clinical evidence of EHV1 and EHV4 was not detected; however, transportation has led to increased virus reactivation of EHV2 and EHV5, shedding and transmission, 

The gene B (gB) was chosen as it is a highly conserved gene among all herpesvirus genomes [59]; furthermore, it is involved in the fusion of viral and cellular membranes, leading to virus entry into the host cell. Following initial binding to the host receptors, membrane fusion is mediated by fusion machinery composed of at least the gB, gH and gL gene homologues, essential for herpesvirus infectivity, mediating the fusion between the virion envelope and the outer nuclear membrane during virion exit out of the infected cell [60].

Partial sequences of the gB gene of EHV strains were used to compare their phylogenetic relationships with each other and other sequences from GenBank. The phylogenetic analyses of gB genes indicated that the Tunisian EHV isolates display genetic diversity within the equid gammaherpesviruses. This is in agreement with previous studies conducted elsewhere [35,42,61], and such genetic diversity may influence the spread of these infections with repeated infections and viral recombination in horses [46].

It is known that EHV2 and EHV5 exhibit a high degree of genetic heterogeneity [42,61,62]. Thus, our results demonstrated that genetic diversity was higher amongst Tunisian EHV2 than EHV5 sequences; similar findings were reported in Turkey [35]. Such high sequence heterogeneity has been demonstrated among EHV2 strains, and a single horse can be infected simultaneously with more than one virus strain [62]; multiple infections of one horse with several different genotypes of EHV2 have also been reported [63]. There was some correlation between genomic variation and cross-neutralization data, which suggests that immune selection may be the major force behind EHV2 heterogeneity [64,65]. 

We reconstructed the evolutionary history of available strains of equid gammaherpesviruses along with the most likely origin of the analyzed sequences, the most relevant countries that have been recognized as important centers of virus diversification and the most important spread routes. 

Equid gammaherpesviruses are widely distributed around the world. Our phylogeographic results suggested Australia as the most likely origin for all currently available sequences. From there, EHV2 strains diversified in several directions, starting especially in USA and Iceland. For EHV5 strains, virus spreading occurred first in Iceland and later through the Americas and Asian countries, such as China in 2008. Such virus spread could be directly related to intensive horse movements for competition events. Indeed, international horse movements for competitions, breeding or sales are essential for the horse industry and one of the most important factors in the spread of various pathogens [66].

The present study described the detection and genetic characterization of equid gammaherpesviruses isolates from Tunisian horses and provided a detailed epidemiological picture of these viruses in Tunisia. The partial sequences of gB genes of the Tunisian EHV2 isolates exhibited a high degree of genetic heterogeneity. Phylogeographic analyses demonstrated that EHV2 and EHV5 are introduced into the country coming from the USA and Iceland. Nevertheless, more work is needed to amplify and characterize all EHV1 suspected isolates. Limitations of this study included a lack of amplification of positive EHV1 samples, which would provide valuable information about the pathogenicity of the virus and its involvement in respiratory disease, abortion and neurological disorders. Further research work is needed to outline the possible importance of genetic variations as they are closely related to gammaherpesviruses evolution and pathogenesis.

## 4. Materials and Methods

### 4.1. Sample Collection

A total number of 298 samples (288 swabs: 277 nasal, 10 vaginal, 1 tracheal and 10 organs (from aborted fetuses)) were collected from horses showing various signs of respiratory illness (*n* = 230), including coughing, nasal discharge, fever, dyspnea, neurological signs (*n* = 5) and from aborted fetuses (*n* = 10). In some cases, nasal swabs were collected from apparently healthy horses in contact with infected horses. All data of the sampled horses are summarized in Table S1. All samples were immediately placed in a cooler box and transported to the Laboratory of Veterinary Epidemiology and Microbiology at the Institute Pasteur of Tunis. Upon arrival, 500 µL of Dulbecco’s modified Eagle medium (DMEM) (PAN Biotech, Aidenbach, Germany) containing 5% antibiotics were added to each swab and vortexed in situ for 1 min to release the virus. Organs were washed in PBS (pH 7.2) and homogenized in a blender in the presence of DMEM, containing 5% antibiotics. All mixtures were clarified by centrifugation at 1500 rpm for 15 min, passed through a 0.22 µm filter and stored at −80 °C.

### 4.2. DNA Extraction and qPCR Amplification

Total viral DNA was extracted from 200 µL of each collected sample using a QIAamp cador Pathogen Mini Kit (Qiagen, Hilden, Germany), according to the manufacturer’s instructions. Nucleic acids were eluted in a final volume of 50 μL and stored at −80 °C until used. Quantitative PCR was performed with LightCycler 480 Software (Roche Life Science). Primers and probes specific to the glycoprotein B (gB) genes of EHV1, 2, 4 or 5 were used as described in Table 1. Each test was used as a simplex assay in a total volume of 25 µL containing 2XTaqman Universal PCR Master Mix (Applied Biosystems, Carlsbad, CA, USA). Each reaction mix consisted of 5 µL extracted DNA, 12.5 µL (Taqman Universal PCR Master Mix (2X)), 0.5 µL probe, 1 µmol forward and reverse primers (1 µL each), and 5 µL nuclear free water for each sample, were analyzed. The cycling conditions for the thermal profile were as follows: holding for 2 min at 95 °C, followed by 45 cycles of amplification of 3 s at 95 °C and 30 s at 60 °C and then holding for 1 min at 60 °C. Each viral PCR test included a negative control made of distilled water and a specific positive control of each viral DNA to be amplified (Table 3).

### 4.3. Conventional PCR and Sequencing

PCR amplification was performed using specific primers of EHV2 and EHV5, as summarized in (Table 3). Each PCR reaction was performed using a KAPA Taq PCR kit with 2 µL (10 µM) of each of the two selected primers, 5 μL of 10XKAPA Taq buffer, 1 μL of dNTP mix (0.2 mM), 0.2 μL of Taq polymerase (0.02 U/μL), 2 μL of DNA extract and nuclease-free water in a final volume of 50 μL. Amplification was carried out in a Bio-Rad T100 thermal cycler (Hercules, CA, USA), using the following reaction steps: an initial denaturation step of 95 °C for 5 min, followed by 40 cycles of amplification, using denaturation at 95 °C for 30 s, annealing at 61 °C for 45 s, extension at 72 °C for 1 min and final extension at 72 °C for 10 min. As a negative control, nuclease-free water was used. The final specific PCR products were visualized using 1.5% agarose gel electrophoresis. The gels were examined for specific size bands using a Gel Doc 2000 system (Bio-Rad). Samples were sequenced using ABI BigDye^®^ Terminator v3.1 (Applied Biosystems) on an ABI PRISM^®^ 3100 Genetic Analyzer (Applied Biosystems).

### 4.4. Statistical Analyses

Statistical analyses were performed using MedCalc Statistical Software v20.106, available online (https://www.medcalc.org/calc/relative_risk.php, accessed on 5 December 2021). The association between the increased infection risks with EHVs within multiple variables was evaluated by calculating the relative risk (RR) with a 95% confidence interval (CI). For all comparisons, a *p*-value ≤ 0.05 was considered to be statistically significant.

### 4.5. Phylogeographic Analysis

The investigated nucleotide sequences (gB gene) of EHV2 and EHV5 were submitted to GenBank and received the following accession numbers: OL859490-OL859530; their metadata are summarized in Table S2. After performing a BLASTN of these sequences against GenBank at NCBI (https://blast.ncbi.nlm.nih.gov, accessed on 23 November 2021), from the best matches, a selection of sequences per year and per country were extracted (*n* = 68). 

Prior to the phylogenetic reconstruction with the PhyML v3.0 [70] program (Kimura two-parameter distance), a multiple sequence alignment was obtained using Bioedit v7.2.5.0 [71]. The Kimura two-parameter distance model was selected through the use of the SMS tool (http://www.atgc-montpellier.fr/sms/, accessed on 5 December 2021).

The generated Maximum Likelihood tree was used as an input for the TempEST v1.5.4 program [72]. Unfitted to the clock-likeness, the outlier sequences, indicated by the root-to-tip regression plot, were removed from our final dataset. To construct a Maximum Clade Credibility (MCC) phylogenetic tree, a Bayesian Markov Monte Carlo chain sampling was performed using BEAST v.1.8.4 software [73]. The location trait was analyzed as a discrete trait diffusion model to track the evolutionary history of equid herpesviruses in Tunisia. Quantification of transition events between different locations was performed by applying a Bayesian Stochastic Search Variable Selection (BSSVS) model with a symmetrical discrete trait substitution model and a strict clock assumption. The uncorrelated relaxed clock model and the skyline tree model were inferred in our Bayesian analysis (500 million iterations and sampling every 10,000 states).

Most of the parameters presented satisfactory Effective Sampling Size (ESS > 200). The MCC tree was generated by TreeAnnotator v1.8.4 [73] after removing 10% burn-in and then visualized in FigTree v.1.4.3 (http://tree.bio.ed.ac.uk/software/figtree/, accessed on 3 December 2021). Using the same program, the time of the most recent common ancestor (tMRCA) was determined along with their 95% highest posterior density (95% HPD). From the MCC tree, the SpreaD3 program [74] produced a KML file (supplementary) that was used to display the geographic and temporal data in Google Earth Pro (https://www.google.com/earth/versions/, accessed on 5 December 2021). Only the transitions tracking the evolutionary history of equid herpesviruses in Tunisia were displayed on the 3D map in the Google Earth Pro program. The Bayes Factor was also computed with SpreadD3.

## Figures and Tables

**Table 1:** Incidence of equid herpesvirus EHV1, EHV2 and EHV5 in Tunisia.

Infection	Virus	Negative	Positive	% Infection
Total	EHV1 EHV2 EHV5	287	11	3.69
		152	146	48.99
		139	159	53.35
Unique infection	EHV1 EHV2 EHV5	293	5	1.67
		249	49	16.44
		238	60	20.13
Co-infection	EHV1, EHV2 EHV1, EHV5 EHV2, EHV5	297	1	0.33
		294	4	1.34
		204	94	31.54
Triple infection	EHV1, EHV2, EHV5	297	1	0.33
Global incidence	EHV1, EHV2, EHV4, EHV5	84	214	71.81

**Table 2:** Frequency of PCR detection of equid herpesvirus EHV1, EHV2 and EHV5 (*n* = 298) depending on age, sex, breed, region, activity, season and clinical status, at the time of sampling, in Tunisia.

		EHV1				EHV2				EHV5			
Category	N° Tested	N° Positive (%)	RR	95% CI	*p*-Value	N° Positive (%)	RR	95% CI	*p*-Value	N° Positive (%)	RR	95% CI	*p*-Value
Age. Year													
<5	170	6 (54.5)	0.9035	0.2820–2.8951	0.8644	91 (62.3)	1.2458	0.9762–1.5898	0.0773	103 (64.8)	1.3849	1.0994–1.7445	0.0057
05–10	82	1 (9.1)	0.2634	0.0343–2.0256	0.1999	44 (30.1)	1.1363	0.8888–1.4527	0.3079	37 (23.3)	0.7989	0.6124–1.0422	0.0978
>10	30	3 (27.3)	3.3500	0.9388–11.9544	0.0625	8 (5.5)	0.5179	0.2829–0.9481	0.0329	15 (9.4)	0.9306	0.6398–1.3535	0.7066
Sex													
Male	143	2 (18.2)	0.2409	0.0529–1.0961	0.0656	76 (52.1)	1.1768	0.9332–1.4840	0.1688	90 (56.6)	1.4138	1.1390–1.7549	0.0017
Female	139	8 (72.7)	3.0504	0.8253–11.2743	0.0945	67 (45.9)	0.9701	0.7687–1.2244	0.7984	65 (40.9)	0.791	0.6351–0.9851	0.0363
Breed													
Arabian thoroughbred	186	4 (36.4)	0.3441	0.1030–1.1492	0.0829	101 (69.2)	1.3515	1.0404–1.7557	0.024	102 (64.2)	1.0775	0.8614–1.3479	0.5132
English thoroughbred	46	1 (9.1)	0.5478	0.0718–4.1775	0.5615	23 (15.8)	1.0244	0.7473–1.4043	0.881	29 (18.2)	1.2221	0.9503–1.5716	0.1181
BAB	57	5 (45.5)	0.3986	0.1994–0.7967	0.0092	19 (13.01)	1.9211	1.1632–3.1727	0.0108	22(13.83)	1.8198	1.1235–2.9476	0.0150
Others	9	1(9.1)	0.3066	0.0419–2.2426	0.2442	3 (2.05)	1.9211	0.4895–7.5388	0.3493	6(3.77)	0.5719	0.1458–2.2443	0.4231
Activity													
Race	226	5 (45.5)	0.2655	0.0835–0.8442	0.0246	125 (85.6)	1.8963	1.2987–2.7690	0.0009	127 (79.9)	1.2644	0.9530–1.6775	0.1039
Leisure	25	1 (9.1)	1.0920	0.1457–8.1863	0.9318	9 (6.2)	0.7174	0.4198–1.2259	0.2244	16 (10.1)	1.2218	0.8917–1.6742	0.2126
Breeding	24	4 (36.4)	6.5238	2.0544–20.7165	0.0015	7 (4.8)	0.5749	0.3049–1.0842	0.0872	7 (4.4)	0.5258	0.2793–0.9896	0.0463
Show jumping	7	-	1.5870	0.1022–24.6503	0.7414	2 (1.4)	0.5774	0.1779–1.8735	0.3604	5 (3.1)	1.3497	0.8344–2.1832	0.2216
Season													
Winter	129	3 (27.3)	0.4913	0.1330–1.8153	0.2865	69 (47.3)	1.174	0.9324–1.4781	0.1723	68 (42.8)	0.9848	0.7940–1.2213	0.8887
Spring	84	2 (18.2)	0.5661	0.1249–2.5660	0.4606	41 (28.1)	0.9948	0.7685–1.2877	0.9683	45 (28.3)	1.0056	0.7948–1.2725	0.9626
Summer	63	1 (9.1)	0.3730	0.0487–2.8595	0.3426	32 (21.9)	1.0471	0.7942–1.3805	0.7444	40 (25.2)	1.2538	1.0004–1.5715	0.0496
Autumn	22	5 (45.5)	10.4545	3.4642–31.5507	<0.0001	4 (2.7)	0.3534	0.1446–0.8639	0.0226	6 (3.8)	0.492	0.2466–0.9814	0.0441
Clinical signs													
Yes	245	6 (54.5)	0.2596	0.0823–0.8191	0.0214	126 (86.3)	1.3629	0.9446–1.9664	0.0979	137 (86.2)	1.3471	0.9604–1.8896	0.0844
No	43	4 (36.4)	3.3887	1.0358–11.0861	0.0436	18 (12.3)	0.8339	0.5744–1.2108	0.3398	21 (13.2)	0.9024	0.6513–1.2504	0.5372
Governorate													
North	256	10(90.1)	0.9429	0.7776–1.1433	0.5496	130 (89.04)	0.9310	0.8492–1.0206	0.1273	139(87.42)	0.9628	0.8772–1.0568	0.4255
South	30	1(9.1)	1.1115	0.1662–7.4342	0.9132	13(8.90)	1.2561	0.6329–2.4930	0.5145	13(8.17)	1.4958	0.7538–2.9684	0.2495
Imported	12	-	0.9599	0.0598–15.4164	0.9769	3 (2.1)	0.5	0.1864–1.3414	0.1686	7 (4.4)	1.0976	0.6721–1.7924	0.7098

RR: Relative risk; 95% CI: Confidence interval, BAB: Barb/Arab-Barb.

**Table 3:** Primers and probes used for amplification and sequencing of gB of equid herpesvirus.

Amplification	Virus	Region	Primers and Probes (5′-3′)	Size	References
Real time PCR	EHV1	gB	FW: GGGGTTCTTAATTGCATTCAGACC	106 bp	[67]
			RV: GTAGGTGCGGTTAGATCTCACAAG		
			FAM TCTCCAACGAACTCGCCAGGCTGTACC BHQ1		
	EHV2	gB	FW: GTGGCCAGCGGGGTGTTC	78 bp	[47]
			RV: CCCCCAAAGGGATTYTTGAA		
			FAM CCCTCTTTGGGAGCATAGTCTCGGGG TAMRA		
	EHV4	gB	FW: TAGCAAACACCCACTAATAATAGCAAG	78 bp	[67]
			RV: GCTCAAATCTCTTTATTTTATGTCATATGC		
			HEXCGCAACAGGAACTCACTTCAGAGCCAGC BHQ1		
	EHV5	gB	FW: AACCCGCCGTGCATCA	66 bp	[47]
			RV: AGGCGCCACACACCCTAA		
			FAMACAACACCACCAACCCCTTTCTGCTG TAMRA		
Conventional PCR	EHV2	gB	FW: GCCAGTGTCTGCCAAGTTGATA	444 bp	[68]
			RV: CATGGTCTCGATGTCAAACACG		
	EHV5	gB	FW: ATGAACCTGACAGATGTGCC	293 bp	[69]
			RV: CACGTTCACTATCACGTCGC		

FW: Forward; RV: Reverse; FAM, HEX: Fluorophore; BHQ1, TAMRA: Quencher.

## Data Availability

Not Applicable.

## Supplementary Materials

The following supporting information can be downloaded at: https://www.mdpi.com/article/10.3390/pathogens11091016/s1, Table S1: data information of sampled horses; Table S2: data information of EHV2 and EHV5 Tunisian isolates, File SF1: KML file of Tunisian EHVs.
